# Characterization of *Erysipelothrix rhusiopathiae* Isolates from Diseased Pigs in 15 Chinese Provinces from 2012 to 2018

**DOI:** 10.3390/microorganisms9122615

**Published:** 2021-12-17

**Authors:** Chao Wu, Changjie Lv, Ya Zhao, Weifeng Zhu, Liang Liu, Ting Wang, Chao Kang, Ying Yang, Xiaomei Sun, Qiang Zhang, Meilin Jin

**Affiliations:** 1 State Key Laboratory of Agricultural Microbiology, Huazhong Agricultural University, Wuhan 430070, China; wudlnchao123@163.com (C.W.); lcjandjt@163.com (C.L.); zya9811@163.com (Y.Z.); zhuweifeng8@126.com (W.Z.); robin2371@163.com (L.L.); wangting917@163.com (T.W.); kangchaosuper@163.com (C.K.); f107yy@163.com (Y.Y.); sunxm320@126.com (X.S.); 2 College of Veterinary Medicine, Huazhong Agricultural University, Wuhan 430070, China; 3 College of Biomedicine and Health, Huazhong Agricultural University, Wuhan 430070, China

**Keywords:** *Erysipelothrix rhusiopathiae*, multilocus sequence typing, virulence, antimicrobial susceptibility

## Abstract

*Erysipelothrix rhusiopathiae* can cause erysipelas in animals and erysipeloid in humans. Since its recurrence in 2012, swine erysipelas has caused serious losses within the pig industry in China. The aim of this study was to perform multilocus sequence typing and understand the virulence and antimicrobial susceptibility of *E. rhusiopathiae* isolates in China. Multilocus sequence typing (MLST) of a total of 120 strains was performed, and as a result, three different sequence types were identified, of which ST48 was the main one. Five isolates of each MLST type were randomly selected to be used to challenge mice. ST48 was associated with a higher virulence. Antimicrobial susceptibility was tested using a microdilution technique and, to analyze the resistance mechanism, six strains were selected for genome sequencing. A comparison of the six genomes indicated the presence of a suspected macrolide resistance gene, namely, *Erm(A)-like*, in erythromycin-resistant strains, which increased the minimum inhibitory concentration (MIC) of erythromycin against *E. coli* C600 at least four-fold. In addition, three mutations (*gyrA*86T-I, *gyrA*90D-N, and *parC*81S-I) were observed in the quinolone resistance-determining regions (QRDRs) of *gyrA* and *parC* in quinolone-resistant strains. After the *gyrA* gene with the 86T-I mutation or the *parC* gene with the 81S-I mutation was transfected into *E. coli* C600, the MIC of enrofloxacin against this strain increased at least two-fold. Our findings provide a theoretical basis for developing antibacterial drugs and may contribute to the clinical prevention and control of *E. rhusiopathiae*.

## 1. Introduction

The genus *Erysipelothrix* is divided into four categories: *Erysipelothrix rhusiopathiae* (serotype 1a, 1b, 2, 4, 5, 6, 8, 9, 11, 12, 15, 16, 17, 19, 21, and N), *Erysipelothrix tonsillarum* (serotype 3, 7, 10, 14, 20, 22, and 25), *Erysipelothrix* sp. strain 1 (serotype 13), and *Erysipelothrix* sp. strain 2 (serotype 18) [1,2]. *E. rhusiopathiae* is a small, Gram-positive, slender, straight, rod-shaped bacterium that causes erysipelas in swine and many other animals [3,4,5,6,7,8]. Swine erysipelas can cause pyrexia, lameness, characteristic diamond skin lesions, and even sudden death in growing and adult swine [9]. It occurs worldwide, causing huge economic loss [10,11,12]. In addition, *E. rhusiopathiae* is also a zoonotic pathogen that can cause a skin disease called erysipeloid in humans.

In recent years, methods such as *Spa* typing, multilocus sequence typing (MLST), pulse-field gel electrophoresis (PFGE), and whole-genome analysis have been widely used to analyze the genotype of *E. rhusiopathiae* [2,12,13,14,15]. MLST was established by amplifying and sequencing the seven housekeeping genes (*gpsA*, *recA*, *purA*, *pta*, *prsA*, *galK*, and *ldhA*) of 165 *E. rhusiopathiae* strains isolated in Europe and the U.S.A. However, no isolates from China were involved; thus, MLST of *E. rhusiopathiae* in China has not been performed yet.

Studies in recent years have revealed the serious drug resistance problems of *E. rhusiopathiae* on a global scale [16,17,18]. The *tet(M)* gene found in tetracycline-resistant *E. rhusiopathiae* has 99% similarity to the *tet(M)* gene in *Enterococcus faecalis* [19]. Some clinical isolates are highly resistant to pleuromutilin, lincosamide, and streptogramin A (called the PLSA type). A large number of drug resistance genes have been detected in PLSA-type strains, and sequencing analysis has found a multi-drug resistance gene cluster (*orf1–aadE–apt–spw–lsa(E)–lnu(B)–rec–orf2–orf1–aadE–sat4–aphA3*) [20]. In addition, a 3749 bp plasmid, pER29, was identified in *E. rhusiopathiae* isolated in China, and this plasmid carries the macrolide resistance gene *erm(T)* [21].

Since the 1990s, due to the widespread use of antibiotics and the improvements made in pig farm management, swine erysipelas has been effectively controlled in China. However, since 2012, swine erysipelas has broken out in pig farms in many provinces, which has posed a serious threat to the Chinese pig industry [22]. Considering this, the present study performed MLST and analyzed the antimicrobial susceptibility of 120 *E. rhusiopathiae* clinical strains isolated from diseased pigs from 2012 to 2018 in China. Certain strains were selected for mouse infection experiments to prove the association between virulence and MLST sequence types, as well as to determine the resistance mechanisms using genome sequencing.

## 2. Materials and Methods

### 2.1. Bacterial Isolates

A total of 120 *E. rhusiopathiae* clinical strains were isolated from diseased pigs suspected to be infected with swine erysipelas (septicemia, urticaria, arthritis, endocarditis, and lymphadenitis). These pigs were from unrelated large-scale pig farms in 15 provinces of China and were analyzed between 2012 and 2018 (Table S1). Some of these farms used swine erysipelas-associated vaccines, but none of the pigs were previously infected with *E. rhusiopathiae*. Disease material such as blood, spleen, lymph node, and joint fluid was collected from the diseased pigs suspected of swine erysipelas infection, inoculated in tryptic soy broth (BD, Franklin Lake, NJ, USA) medium containing 10% bovine serum (Sijiqing, Hangzhou, Zhejiang, China), and incubated for 24 h at 37 °C [23]. The isolates were identified as *E. rhusiopathiae* by 16srDNA sequencing. 

### 2.2. MLST Analysis

The total DNA of the clinical isolates was extracted as described previously (18). Using the previously reported amplification method [2], the extracted DNA was used as a template to amplify 7 housekeeping genes (*gpsA*, *recA*, *purA*, *pta*, *prsA*, *galK*, and *ldhA*) of *E. rhusiopathiae*. The sequencing results were compared with the reported sequence types to determine the sequence types of the 120 clinical isolates. If the sequencing result was inconsistent with the reported sequence types, a new sequence type was identified.

### 2.3. Mouse Infection Experiment

Five isolates of each MLST type were randomly selected to be used to challenge mice at 7 gradient doses (320, 160, 80, 40, 20, 10 CFU, and 5 CFU), and each dose was tested in five 4-week-old ICR mice via subcutaneous injection, with PBS as the negative control. The clinical signs and death of the mice were observed once a day for 14 days. The 50% lethal dose (LD50) was calculated by the Reed–Muench method [24]. The LD50 among the different MLST isolates was compared using one-way ANOVA by GraphPad Prism 8 [25]. A *p* value of 0.05 was considered to be statistically significant. The protocol for the in vivo experiments was approved by the Scientific Ethics Committee of Huazhong Agricultural University (Approval Number: HZAUMO-2021-0178).

### 2.4. Antimicrobial Susceptibility Assay

MICs of tetracycline, erythromycin, clindamycin, ciprofloxacin, enrofloxacin, meropenem, and cefotaxime were calculated by the microdilution technique to determine the resistance phenotypes of 120 clinical isolates. *Streptococcus pneumoniae* ATCC 49619 served as the quality control strain. The breakpoint of the minimum inhibitory concentration (MIC) was established by the Clinical and Laboratory Standards Institute (CLSI, 2010).

### 2.5. Genome Sequencing and Comparative Genomic Analysis

Based on the results of the antimicrobial susceptibility assay, 6 strains (B18, B52, B2, SE25, SE27, and SE-RD strains) with representative drug resistance spectra were selected for whole-genome sequencing (Table S2). High-quality genomic DNA was extracted using a modified CTAB method. Qualified genomic DNA was fragmented using G-tubes (Covaris) and then end-repaired to prepare SMRTbell DNA template libraries (with a fragment size > 10 kb selected using a bluepippin system) according to the manufacturer’s instructions (Pacific Biosciences). Library quality was analyzed by Qubit, and the average fragment size was estimated using an Agilent 2100 Bioanalyzer (Agilent, Santa Clara, CA, USA). Whole-genome sequencing was performed on the PacBio Sequel II system (Frasergen Bioinformatics Co., Ltd., Wuhan, China). The PacBio reads were de novo assembled using Microbial Assembly (smrtlink8), HGAP4 [26], and Canu (v1.6) [27]. The depth of genome coverage was analyzed using the pbalign (BLASR, v0.4.1) tool [28]. The genomes of strains B18, B52, B2, SE25, SE27, and SE-RD were annotated using Glimmer (v3.02) [29].

BLASTN (default parameters) was used for genome comparison, and the obtained results were imported into the Artemis Comparison Tool for visualization [30]. The sequences were ordered according to the visualization results. After ordering, the sequences were revisualized with ACT software. Six genome sequences were aligned with each other, and BLAST (basic local alignment search tool) was applied to the genes in the mismatched areas to search for suspected antibiotic resistance genes.

### 2.6. Detection of the Distribution of Drug Resistance Genes and Mutants

The total DNA of each strain was extracted as a template for PCR amplification [31]. To analyze the distributions of drug resistance genes and mutants in 120 isolates, the suspected macrolide resistance gene *Erm(A)-like*, the reported macrolide resistance gene *Erm(T)*, the tetracycline resistance gene *tet(M)*, and *lsa(E)* (representing the *lsa(E)*-carrying multiresistance gene cluster) were amplified by PCR, and the QRDRs of *gyrA* and *parC* were amplified and sequenced. The primers are shown in Table S3. The 50 µL PCR reaction solution contained 1 µL of DNA, 1 µL of each primer, 25 µL of PrimeSTAR Max Premix (2×) (Takara Biomedical Technology (Beijing) Co., Ltd., Beijing, China), and 22 µL of deionized distilled water.

### 2.7. Analysis of the Contribution of Suspected Drug Resistance Genes and Mutants

The wild-type genes *gyrA* and *parC* from the sensitive strain SE-RD, the suspected macrolide resistance gene *Erm(A)-like*, the mutated *gyrA* (90D-N) and *parC* (81S-I) from the resistant strain B18, and *gyrA* (86T-I) from the resistant strain B2, were separately cloned into pSET2 plasmids. The recombinant plasmids were transfected into *E. coli* C600, as described previously [32]. The primers used are presented in Table S3. The recombinant strains were subjected to an antimicrobial susceptibility assay by the method mentioned in Section 2.3. The C600 (pSET2) strain (transformed with the pSET2 plasmid) and wild-type *E. coli* C600 were used as controls.

### 2.8. Accession Numbers

The genome sequences of strains B18, B52, B2, SE25, SE27, and SE-RD were uploaded to NCBI GenBank with the accession numbers PRJNA750282, PRJNA750613, PRJNA750854, PRJNA750858, PRJNA750871, and PRJNA750617.

### 2.9. Statistical Analysis

The LD50 of the different MLST isolates was compared using one-way ANOVA by GraphPad Prism 8; a *p* value of 0.05 was considered to be statistically significant.

## 3. Results

### 3.1. MLST and Virulence o thef Isolates

**Figure 1:**
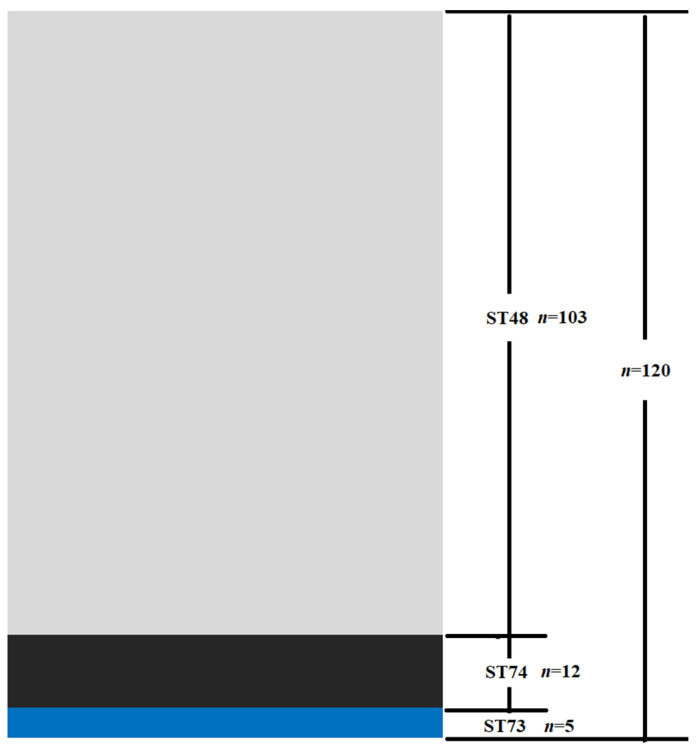
Distribution of multilocus sequence types of 120 strains isolated in China. Three sequence types were identified, with the number of Sequence Type 48 (ST48), Sequence Type 73 (ST73), and Sequence Type 74 (ST74) strains being 103, 5, and 12 respectively.

In total, 7 housekeeping genes (*gpsA*, *recA*, *purA*, *pta*, *prsA*, *galK*, and *ldhA*) from 120 clinical strains were amplified and sequenced. One new allele was discovered separately for *gpsA* and *recA.* Based on new alleles*,* two new sequence types were identified and named ST73 and ST74. Moreover, 3 sequence types (two new types and one known type) were identified from the 120 strains. The largest number of strains (*n* = 103) belonged to ST48, followed by ST74 (*n* = 12) and ST73 (*n* = 5). The distribution of MLST of the 120 isolates is shown in Figure 1.

**Figure 2:**
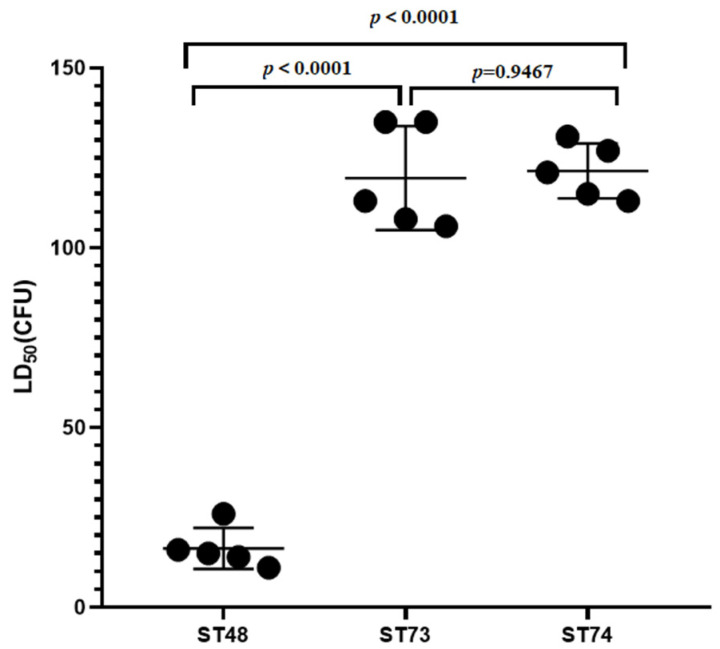
Lethality dose 50 (LD_50_) of isolates and their association with Multilocus Sequence Type (MLST) (one-way ANOVA by GraphPad Prism 8; a *p* value of 0.05 was considered to be statistically significant).

The LD50 of *E. rhusiopathiae* ranged from 11CFU to 135CFU. The LD50 of ST48 isolates (11CFU, 14CFU, 15CFU, 16CFU, and 26CFU) was significantly different from that of ST73 (106CFU, 108CFU, 113CFU, 135CFU, and 135CFU) and ST74 (113CFU, 115CFU, 121CFU, 127CFU, and 131CFU) (ST48 vs. ST73: *p* < 0.0001; ST48 vs. ST74: *p* < 0.0001), whereas there was no significant difference in LD50 between ST73 and ST74 isolates (ST73 vs. ST74: *p* = 0.9467). Compared to ST73 and ST74, ST48 was associated with a higher prevalence of highly virulent isolates (Figure 2).

### 3.2. Antimicrobial Susceptibility

The MICs of 7 antibiotics against the 120 strains are listed in Table 1. All strains were sensitive to meropenem and cefotaxime, and the resistance rates to tetracycline, erythromycin, clindamycin, ciprofloxacin, and enrofloxacin were 50.8%, 53.3%, 70.0%, 91.7%, and 82.5%, respectively. The drug resistance spectra of the 7 antibiotics against the 120 strains are shown in Table S4. One strain was sensitive to all seven antibiotics, and 44.2% of the strains were resistant to five antibiotics, namely, ciprofloxacin, enrofloxacin, clindamycin, tetracycline, and erythromycin.

### 3.3. Genome Sequencing and Comparative Genomic Analysis

Genome and assembly statistics of each strain are summarized in Table S5. The number of predicted coding sequences (CDSs) of the six sequenced genomes ranged from 1661 to 1840. The genome of strain B18 carried the greatest number of rRNA loci. The guanine and cytosine (GC) content ranged from 36.21% to 36.37%. Several genome islands and prophages were identified, with the number of genome islands ranging from three to seven, and that of prophages from zero to two.

**Figure 3:**
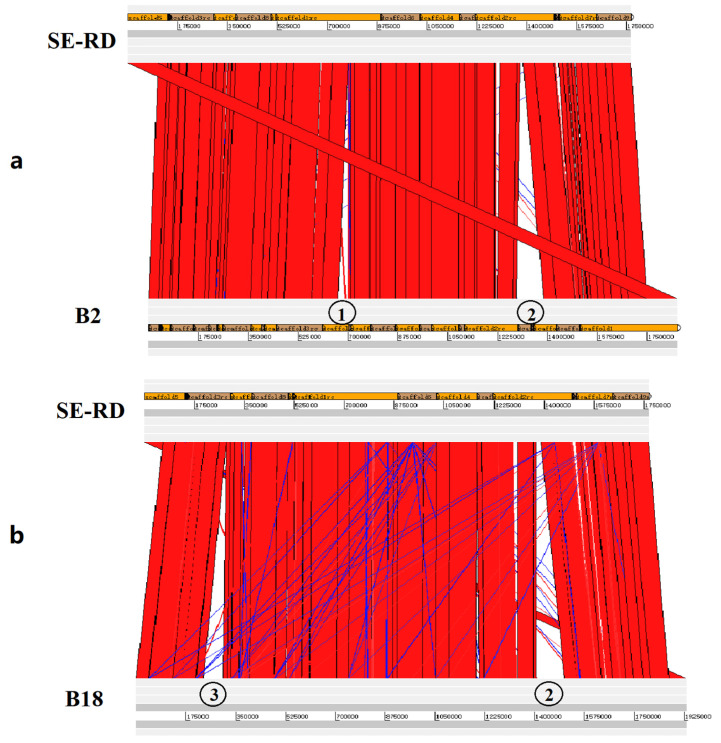
Comparative genomic analysis of different strains. Alignment between SE-RD and B2 (**a**) and between SE-RD and B18 (**b**). Alignment against blastn (default parameters); visualization with Artemis Comparison Tool (http://sanger-pathogens.github.io/Artemis/ACT/,accessed on 11 August 2020). ① A 35Kb mismatched area (no suspected drug resistance genes) found in B2; ② a 77 Kb mismatched area (*lsa(E)*-carrying multiresistance gene cluster) found in B2 and B18; ③ a 70 Kb mismatched area (containing the *Erm(A)-like* gene) found in B18.

Six genome sequences were aligned with each other (Figures S1–S15), and three mismatched areas were found in the genomes (Figure 3). The first one (about 35 Kb) was found only in strain B2, and no suspected drug resistance genes were found. The second one (about 70Kb) was found in B18 and B52, and in this area, a suspected macrolide antibiotic resistance gene, *Erm(A)-like*, was also found. The amino acid sequence of *Erm(A)-like* exhibited 59% similarity to that of *Erm(T)* (the only macrolide antibiotic resistance gene reported in *E. rhusiopathiae*) [21]. The third one (about 77Kb) was found in B18, B52, B2, and SE25, and it was the reported *lsa(E)*-carrying multiresistance gene cluster (ACCESSION: KP339868) containing *lsa (E)*, *spw*, *lnu (B)*, *aadE, aphA3,* and other drug resistance genes [20].

### 3.4. Distribution of Drug Resistance Genes and Resistance-Conferring Mutants

The distribution of *Erm(A)-like* and the known *Erm(T)* in clinical isolates is presented in Table S6. *Erm(A)-like* was detected in all 64 erythromycin-resistant strains, but it was undetected in all 56 erythromycin-sensitive strains. *Erm(T)* was not detected in the bacteria. These results indicated that the *Erm(A)-like* gene was widely distributed in the erythromycin-resistant strains of *E. rhusiopathiae* isolated in China.

The 120 *E. rhusiopathiae* isolates fell into 3 *gyrA*–*parC* genotypes according to the QRDRs of *gyrA* and *parC* (Tables S7 and S8). Of these 120 isolates, 5 strains, including the sequenced quinolone-sensitive strain SE-RD, belonged to the genotype *gyrA*T86–*gyrA*D90–*parC*S81. The MIC of ciprofloxacin for these five strains was 0.06 μg/mL, and that of enrofloxacin was 0.06 μg/mL or 0.125 μg/mL. The 110 isolates, including the sequenced B18, B52, SE25, and SE27, belonged to the genotype *gyrA*T86–*gyrA*N90–*parC*I81. Five isolates, including the sequenced B2, belonged to genotype *gyrA*I86–*gyrA*N90–*parC*I81. The MIC of ciprofloxacin for the last two genotype strains was 1–8 μg/mL, and that of enrofloxacin was 0.5–16 μg/mL. Our results indicate that the three mutants, namely, *gyrA*86T-I, *gyrA*90D-N, and *parC*81S-I, were widely distributed in the quinolone resistance-determining regions (QRDRs) of *gyrA* and *parC* in *Erysipelothrix rhusiopathiae* isolated in China.

The *tet(M)* gene was detected in 45 out of the 120 isolates, with a detection rate of 37.5%. These 45 positive isolates included 35 tetracycline-resistant strains and 10 sensitive strains (Table S9). The *las(E)* gene was detected in 71 of the 120 isolates, with a detection rate of 59.2%. These 71 positive isolates included 68 clindamycin-resistant strains and three sensitive strains (Table S10).

The correlation between the antibiotic resistance genotypes and the phenotypes is shown in Table S11. The results indicated that the relationship between drug resistance phenotype and genotype was very complex.

### 3.5. Contribution of the Newly Identified Drug Resistance Genes and Resistance-Conferring Mutants

The antimicrobial susceptibility of recombinant *E. coli* containing the suspected macrolide resistance gene *Erm(A)-like*, C600 transformed with the pSET2 plasmid, and wild-type *E. coli* C600, was tested. As shown in Table 2, *Erm(A)-like* increased the MIC of erythromycin against *E. coli* C600 at least four-fold.

The antimicrobial susceptibility of recombinant *E. coli* (containing the mutant *gyrA* (90D-N), *parC* (81S-I), and *gyrA* (86T-I) and wild-type *gyrA* and *parC*) C600 transformed with the pSET2 plasmid and that of wild-type *E. coli* C600 was tested. As shown in Table 2, the mutant *gyrA* 86T-I and mutant *parC* 81S-I increased the MIC of enrofloxacin at least two-fold.

## 4. Discussion

*E. rhusiopathiae* is an important zoonotic pathogen that can cause erysipelas in many animals, including pigs, and erysipeloid in humans [3,12]. However, currently, there is insufficient analysis regarding MLST and the mechanisms of virulence and drug resistance of *E. rhusiopathiae*, though swine erysipelas recurred in 2012 in China.

MLST is an important method for bacterial genotyping [24,33,34]. The sequence types of *E. rhusiopathiae* isolated in Europe, Australia, and the United States are mainly ST3, ST9, and ST19 [2]. Our results showed that the sequence type of Chinese isolates is highly conservative and is mainly ST48, which is associated with a higher prevalence of highly virulent isolates.

The abuse of antibiotics can lead to an increase in bacterial resistance [35]. Bacterial resistance is a major global issue that can cause the failure of antibiotic treatments of bacterial diseases; more importantly, a large number of antibiotic-resistant bacteria will eventually endanger human health [36]. Since China has large-scale pig breeding farms, the analysis of drug resistance in *E. rhusiopathiae* isolated from large-scale pig farms throughout the country is conducive to a more scientific and reasonable use of antibiotics to prevent and control *E. rhusiopathiae*. There are many mechanisms of antibiotic resistance, including the alteration of the targets, the inactivation of drugs by hydrolysis or modification, the creation of alternative pathways, efflux pumps, and the inhibition of drug entry into cells [37]. Through comparative genomic analysis of *E. rhusiopathiae* isolates with different drug resistance phenotypes, some new drug resistance genes and mutants were identified. As it is impossible to directly verify the contribution of resistance genes or mutants to bacterial resistance due to the imperfect genetic manipulation of *E. rhusiopathiae*, an alternative model organism is necessary. The nucleotide sequences of *gyrA* and *parC* in *Escherichia coli* are highly similar to those in *E. rhusiopathiae.* Furthermore, *E. coli* C600 is sensitive to erythromycin and quinolone drugs (ciprofloxacin and enrofloxacin). Considering these findings, we chose *E. coli* C600 to further verify the contribution of three mutants of *gyrA* and *parC* to quinolone resistance and of *Erm(A)-like* to erythromycin resistance. Our results indicated that *Erm(A)-like* increased the MIC of erythromycin against *E. coli* C600 at least four-fold and that the mutation of *gyrA* 86T-I or *parC* 81S-I also increased *E. coli* C600 resistance to enrofloxacin at least two-fold. As the MICs for both recombinant strains and wild-type strains were lower than the minimum dilution concentration, the influence of the above two mutants on ciprofloxacin, or that of mutation *gyrA*90D-N on ciprofloxacin or enrofloxacin, could not be determined, indicating that ciprofloxacin and enrofloxacin must be further diluted to determine the MICs for the recombinant and wild-type strains or that a genetic operating system suitable for *E. rhusiopathiae* direct verification should be developed.

The total DNA extraction method can be used to obtain the bacterial genome and possible plasmids together; thus, total DNA can be used as a template for the amplification of *Erm(T)* in plasmids. However, *Erm(T)* was not amplified in the 120 clinical isolates, whereas *Erm(A)-like* was amplified in the erythromycin-resistant strains, indicating that *Erm(A)-like* is more widely distributed in clinical erythromycin-resistant isolates from China than *Erm(T).* Although *Erm(A)-like* was detected in all of the 64 erythromycin-resistant strains, the MIC of erythromycin against these bacteria varied greatly, ranging from 2 to 256 μg/mL, which may be attributed to other unknown erythromycin resistance mechanisms. The *tet(M)* gene was detected in most of the tetracycline-resistant strains rather than in all the strains, and *lsa(E)* was detected in the clindamycin-resistant strains, implying that there might be other resistance mechanisms to tetracycline and clindamycin. Interestingly, *tet(M)* or *lsa(E)* was also detected in some strains sensitive to tetracycline or clindamycin, in spite of a low detection rate, suggesting that there might be some differences in the expression of these resistance genes in clinical isolates. The correlation analysis results indicated that the relationship between drug resistance phenotype and genotype is very complex.

In conclusion, our data revealed the epidemic characteristics of *E. rhusiopathiae* isolates in China from 2012 to 2018 and showed that ST48 was the main sequence type and had the strongest virulence. Furthermore, we also tested the resistance phenotypes of these isolates. The results indicated that *E. rhusiopathiae* isolates from China were highly resistant to tetracycline, erythromycin, clindamycin, and quinolone, suggesting that these classes of antibiotics could be over- or misused in swine production in China. Through further analyzing the resistance mechanism, we not only confirmed that mutations in gyrA and parC were involved in quinolone resistance of *E. rhusiopathiae* strains, but also first discovered the wide distribution of the *Erm(A)-like* gene in *E. rhusiopathiae* strains. These findings could be beneficial to the clinical prevention and control of *E. rhusiopathiae* and the development of antibacterial drugs.

## Figures and Tables

**Table 1:** Distribution of Minimum Inhibitory Concentrations (MICs) of antibiotics against the 120 *E. rhusiopathiae* strains isolated from 2012 to 2018 in China.

Antibiotics	Number of Strains with MIC (μg/mL)															MIC(μg/mL) on the Breakpoint ^a^ of Resistance	Number of Resistant Strains (%)
	≤0.015	≤0.03	≤0.06	≤0.125	0.25	0.5	1	2	4	8	16	32	≥64	≥128	≥256		
TE				1	2	6	29	17	1	3		46	13	2		16	61 (50.8%)
ERY				53	2	1		1	19	20	4	4	6	6	4	2	64 (53.3%)
CLI			31		5	7	9	3	1			13	51			0.5	84 (70.0%)
CIP			5				5	8	96	6						2	110 (91.7%)
EFX			3	2		2	4	10	92	4	3					4	99 (82.5%)
MEM	111	9														1	0 (0%)
CTX		18	77	25												2	0 (0%)

The dark area shows the antibiotic resistance area. ^a^ Breakpoint of MIC was from the Clinical and Laboratory Standards Institute (CLSI, 2010). TE, tetracycline; ERY, erythromycin; CLI, clindamycin; CIP, ciprofloxacin; EFX, enrofloxacin; MEM, meropenem; CTX, cefotaxime.

**Table 2:** Contribution to resistance of suspected drug resistance gene and mutants.

Antibiotic ^a^	Gene or Mutants	MIC (μg/mL)			Times Increase in MIC ^e^
		C600 ^b^	C600(pSET2) ^c^	C600 (Recombinant pSET2) ^d^	
ERY	*erm(A)-like*	64	64	>256	>4
CIP	*wild-type gyrA*	<0.06	<0.06	<0.06	—^f^
	*wild-type parC*			<0.06	—
	*gyrA(90D-N)*			<0.06	—
	*gyrA* *(86T-I)*			<0.06	—
	*par* *C(81S-I)*			<0.06	—
EFX	*wild-type gyrA*	<0.03	<0.03	<0.03	—
	*wild-type parC*			<0.03	—
	*gyrA(90D-N)*			<0.03	—
	*gyrA* *(86T-I)*			0.06	>2
	*par* *C(81S-I)*			0.06	>2

^a^ ERY, erythromycin; CIP, ciprofloxacin; EFX, enrofloxacin. ^b^ Wild-type *E. coli* C600. ^c^ C600 transformed with the pSET2 plasmid. ^d^ C600 transformed with the recombinant pSET2 plasmid containing *Erm(A)-like*, *gyrA (90D-N)*, *gyrA (86T-I)*, or *parC (81S-I)*. ^e^ MIC against C600 (recombinant pSET2) divided by that of C600 (pSET2). ^f^ Since the MICs against the recombinant and wild-type strains were both lower than the minimum dilution concentration, the difference in MIC coulf not be determined.

## Data Availability

Publicly available datasets were analyzed in this study. This data can be found here: PRJNA750282, PRJNA750613, PRJNA750854, PRJNA750858, PRJNA750871, and PRJNA750617.

## Supplementary Materials

The following are available online at https://www.mdpi.com/article/10.3390/microorganisms9122615/s1. Table S1: Background of *E. rhusiopathiae* used in this study. Table S2: A summary of the antimicrobial resistance of whole genome sequencing isolates. Table S3: Primers used in this study. Table S4: Distribution of drug resistant spectra of 120 strains isolated from 2012 to 2018 in China. Table S5: General genome features and assembly statistics of 6 isolates. Table S6: Distribution of *Erm(A)-like* and *Erm(T)* in 120 isolates. Table S7: MICs of ciprofloxacin against 120 isolates and distribution of sequence types of *gyrA-parC*. Table S8: MICs of enrofloxacin against 120 isolates and distribution of sequence types of *gyrA*-*parC*. Table S9: Distribution of *tet(M)* in 120 isolates. Table S10: Distribution of *lsa(E)* gene in 120 isolates. Table S11: Correlation between the antibiotic resistance genotypes and phenotypes of the 120 *E. rhusiopathiae* strains isolated from 2012 to 2018 in China. Figure S1: Alignment between B2 and SE25. Figure S2: Alignment between B2 and SE27. Figure S3: Alignment between B2 and SE-RD. Figure S4: Alignment between B18 and B2. Figure S5: Alignment between B18 and B52. Figure S6: Alignment between B18 and SE25. Figure S7: Alignment between B18 and SE27.Figure S8: Alignment between B18 and SE-RD. Figure S9: Alignment between B52 and B2. Figure S10: Alignment between B52 and SE25. Figure S11: Alignment between B52 and SE27. Figure S12: Alignment between B52 and SE-RD. Figure S13: Alignment between SE25 and SE27. Figure S14: Alignment between SE25 and SE-RD. Figure S15: Alignment between SE27 and SE-RD.
