# Supplementary material for: Characterization of Erysipelothrix rhusiopathiae Isolates from Diseased Pigs in 15 Chinese Provinces from 2012 to 2018
Source: Microorganisms. 2021 Dec 17;9(12):2615. doi: 10.3390/microorganisms9122615 (PMC8704079; doi:10.3390/microorganisms9122615)

Table S1: Background of *E. rhusiopathiae* used in this study

Location of isolation	No. of strains isolated from different diseases					Total
	Septicemia	Urticaria	Arthritis	Endocarditis	Lymphadenitis	
Hubei	13	10	3	1	1	28
Hunan	13	5	7	1		26
Anhui	6	6	5	1	1	19
Henan	3	2	3	1		9
Jiangsu	2	3	2			7
Sichuan		2	3	1		6
Shandong	2	1	2	1		6
Guangxi	2	3	1			6
Jiangxi	1	1		1		3
Guangdong		1	1			2
Yunnan	1		1			2
Liaoning		2				2
Hebei	1	1				2
Zhejiang	1					1
Jilin	1					1
Total	46	37	28	7	2	120

Table S2: A summary of the antimicrobial resistance of whole genome sequencing isolates

antibiotics	strains isolated with MIC (μg/mL)					
	B18	B52	B2	SE25	SE27	SE-RD
CTX	0.06	0.06	0.06	0.06	0.06	0.06
MEM	0.015	0.015	0.015	0.015	0.015	0.015
ERY	8	8	<0.125	<0.125	<0.125	<0.125
CLI	>64	>64	0.25	0.25	<0.06	<0.06
TE	32	32	2	2	0.5	0.5
EFX	4	4	4	4	4	0.125
CIP	4	4	4	4	4	<0.06

A dark area indicates an antibiotic resistance area.

CTX, cefotaxime; MEM, meropenem; ERY, erythromycin; CLI, clindamycin; TE, tetracycline; EFX, enrofloxacin; CIP, ciprofloxacin.

Table S3: Primers used in this study

Functions	Primer sequence (5′–3′) [a] (forward/reverse)
Used to amplify *Erm(A)-like* and construct *E. coli* recombinant plasmid	CGGAATTCATGAACCAGAAAAACCCA/ CGGGATCCTTACTGAAACAATTTGTAACTATTA
Used to amplify *gyrA* and construct *E. coli* recombinant plasmid	CGGAATTCATGGAAGATAATACACAGAGTTATG/ CGGGATCCTTACTCTGCAATTTCTATTTCTT
Used to amplify *parC* and construct *E. coli* recombinant plasmid	CGGAATTCATGTCGCATGATTCATTAA/ CGGGATCCTTATAAATCAAGTTTCAAAGGT
Used to amplify the *Erm(A)-like* and identify its distribution	ACGAACATTACAGGACAGG/ CAATTAAAGGGTGATGACG
Used to amplify the *Erm(T)* and identify its distribution	TATTGAATATGAGAACTTCCAGGTT/ AGTGATATTTTTGAAGGGTGTCTT
Used to amplify the *Tet(M)* and identify its distribution	CGAGGTCAGTCTGAACTTTG/ TTTACAGTCCGTCACATTCC
Used to amplify the *lsa(E)* and identify its distribution	CTTGCAGCTTTATTCCTTAC/ TACTAGACTTACCACTCCCATT
Used to amplify QRDR of *gyrA*	CAGTTCACCGCCGTATTT/ CAACCGCAATCCCAGTA
Used to amplify QRDR of *parC*	TTGGTGAACGGTTTGGTAG/ CTGCGGCAATCCCTGT

[a] Underlined nucleotides denote enzyme restriction sites

Table S4: Distribution of drug resistant spectra of 120 strains isolated from 2012 to 2018 in China

Drug resistant spectrums	Number of strains (%)
Sensitive strain	1(0.8)
CIP	8(6.7)
CIP+EFX	27(22.5)
CLI+ERY	8(6.7)
CIP+CLI+ERY	4(3.3)
CLI+EFX+CIP	11(9.2)
CIP+EFX+CLI+TE	8(6.7)
CIP+EFX+CLI+TE+ERY	53(44.2)

TE, tetracycline; ERY, erythromycin; CLI, clindamycin; CIP, ciprofloxacin; EFX, enrofloxacin.

Table S5: General genome features and assembly statistics of 6 isolates

Characteristic	Description or value for strain					
	B18	B52	B2	SE25	SE27	SE-RD
Size(bp)	1931527	1891093	1855563	1820443	1743896	1765668
G+C (%)	36.36	36.21	36.21	36.26	36.37	36.31
CDSs	1840	1800	1782	1734	1661	1687
Coding density (%)	89.74	89.34	89.93	89.37	89.86	89.92
Avg. length of CDSs(bp)	942.05	938.59	936.42	938.28	943.48	941.15
rRNA Locus	18	6	6	7	6	4
Trna	55	55	55	55	55	55
Genome Islands	5	4	7	6	3	4
Prophage	2	1	1	0	0	1

Table S6: Distribution of *Erm(A)-like* and *Erm(T)* in 120 isolates

distribution of genes	MIC of strains against Erythromycin (μg/mL)											
	≤0.125	0.25	0.5	1	2	4	8	16	32	64	128	≥256
Erm(A)-like	0/53	0/2	0/1	0/0	1/1	19/19	20/20	4/4	4/4	6/6	6/6	4/4
Erm(T)	0/53	0/2	0/1	0/0	0/1	0/19	0/20	0/4	0/4	0/6	0/6	0/4

Table S7: MICs of ciprofloxacin against 120 isolates and distribution of sequence types of *gyrA-parC*

MICs of Ciprofloxacin (μg/mL)	No. of strains	distribution of sequence types of *gyrA-parC*		
		gyrAT_{86}-*gyrA*D_{90}-*parC*S_{81}	*gyrA*T_{86}-*gyrA*N_{90}-*parC*I_{81}	*gyrA*I_{86}-*gyrA*N_{90}-*parC*I_{81}
≤0.06	5	5/5	0/5	0/5
0.125	0	0/0	0/0	0/0
0.25	0	0/0	0/0	0/0
0.5	0	0/0	0/0	0/0
1	5	0/5	5/5	0/5
2	8	0/8	8/8	0/8
4	96	0/96	91/96	5/96
8	6	0/6	6/6	0/6
16	0	0/0	0/0	0/0
32	0	0/0	0/0	0/0
64	0	0/0	0/0	0/0
≥128	0	0/0	0/0	0/0

Table S8: MICs of enrofloxacin against 120 isolates and distribution of sequence types of *gyrA-parC*

MICs of Enrofloxacin (μg/mL)	No. of strains	distribution of sequence types of *gyrA-parC*		
		gyrAT_{86}-*gyrA*D_{90}-*parC*S_{81}	*gyrA*T_{86}-*gyrA*N_{90}-*parC*I_{81}	*gyrA*I_{86}-*gyrA*N_{90}-*parC*I_{81}
≤0.03	0	0/0	0/0	0/0
0.06	3	3/3	0/3	0/3
0.125	2	2/2	0/2	0/2
0.25	0	0/0	0/0	0/0
0.5	2	0/2	2/2	0/2
1	4	0/4	4/4	0/4
2	10	0/10	10/10	0/10
4	92	0/92	90/92	2/92
8	4	0/4	1/4	3/4
16	3	0/3	3/3	0/3
32	0	0/0	0/0	0/0
≥64	0	0/0	0/0	0/0

Table S9: Distribution of *tet(M)* in 120 isolates

MIC of strains against Tetracycline (μg/mL)	≤0.125	0.25	0.5	1	2	4	8	16	32	64	≥128
distribution of *tet(M)*	0/1	0/2	0/6	0/29	7/17	1/1	2/3	0/0	24/46	9/13	2/2

Table S10: Distribution of *lsa(E)* gene in 120 isolates

MIC of strains against Clindamycin (μg/mL)	≤0.06	0.12	0.25	0.5	1	2	4	8	16	32	≥64
distribution of *lsa(E)*	0/31	0/0	3/5	2/7	4/9	2/3	0/1	0/0	0/0	9/13	51/51

Table S11: Correlation between the antibiotic resistance genotypes and phenotypes of the 120 *E. rhusiopathiae* strains isolated from 2012 to 2018 in China

Resistance phenotypes	Number of strains	Resistance genotypes
Sensitive strain	1	*Erm(A)*$^{-}$*tet(M)*$^{-}$*las(E)*$^{-}$*gyrAT86-gyrAD90-parCS81*
CIP	1	*Erm(A)*$^{-}$*tet(M)*$^{+}$*las(E)*$^{-}$*gyrA*I86-*gyrA*N90-*par*CI81
	1	*Erm(A)*$^{-}$*tet(M)*$^{+}$*las(E)*$^{-}$*gyrA*T86-*gyrA*N90-*par*CI81
	6	*Erm(A)*$^{-}$*tet(M)*$^{-}$*las(E)*$^{-}$*gyrA*T86-*gyrA*N90-*par*CI81
CIP+EFX	4	*Erm(A)*$^{-}$*tet(M)*$^{+}$*las(E)*$^{-}$*gyrA*T86-*gyrA*N90-*par*CI81
	3	*Erm(A)*$^{-}$*tet(M)*$^{-}$*las(E)*$^{+}$*gyrA*T86-*gyrA*N90-*par*CI81
	20	*Erm(A)*$^{-}$*tet(M)*$^{-}$*las(E)*$^{-}$*gyrA*T86-*gyrA*N90-*par*CI81
CLI+ERY	1	*Erm(A)*$^{+}$*tet(M)*$^{+}$*las(E)*$^{+}$*gyrA*T86-*gyrA*D90-*par*CS81
	1	*Erm(A)*$^{+}$*tet(M)*$^{-}$*las(E)*$^{+}$*gyrA*T86-*gyrA*D90-*par*CS81
	4	*Erm(A)*$^{+}$*tet(M)*$^{-}$*las(E)*$^{+}$*gyrA*I86-*gyrA*N90-*par*CI81
	2	*Erm(A)*$^{+}$*tet(M)*$^{-}$*las(E)*$^{-}$*gyrA*T86-*gyrA*D90-*par*CS81
CIP+CLI+ERY	4	*Erm(A)*$^{+}$*tet(M)*$^{-}$*las(E)*$^{+}$*gyrA*T86-*gyrA*N90-*par*CI81
CLI+EFX+CIP	3	*Erm(A)*$^{-}$*tet(M)*$^{+}$*las(E)*$^{+}$*gyrA*T86-*gyrA*N90-*par*CI81
	6	*Erm(A)*$^{-}$*tet(M)*$^{-}$*las(E)*$^{+}$*gyrA*T86-*gyrA*N90-*par*CI81
	2	*Erm(A)*$^{-}$*tet(M)*$^{-}$*las(E)*$^{-}$*gyrA*T86-*gyrA*N90-*par*CI81
CIP+EFX+CLI+TE	2	*Erm(A)*$^{-}$*tet(M)*$^{-}$*las(E)*$^{+}$*gyrA*T86-*gyrA*N90-*par*CI81
	1	*Erm(A)*$^{-}$*tet(M)*$^{-}$*las(E)*$^{-}$*gyrA*T86-*gyrA*N90-*par*CI81
	1	*Erm(A)*$^{-}$*tet(M)*$^{+}$*las(E)*$^{+}$*gyrA*T86-*gyrA*N90-*par*CI81
	4	*Erm(A)*$^{-}$*tet(M)*$^{+}$*las(E)*$^{+}$*gyrA*T86-*gyrA*N90-*par*CI81
CIP+EFX+CLI+TE+ERY	15	*Erm(A)*$^{+}$*tet(M)*$^{-}$*las(E)*$^{+}$*gyrA*T86-*gyrA*N90-*par*CI81
	8	*Erm(A)*$^{+}$*tet(M)*$^{-}$*las(E)*$^{-}$*gyrA*T86-*gyrA*N90-*par*CI81
	27	*Erm(A)*$^{+}$*tet(M)*$^{+}$*las(E)*$^{+}$*gyrA*T86-*gyrA*N90-*par*CI81
	3	*Erm(A)*$^{+}$*tet(M)*$^{+}$*las(E)*$^{-}$*gyrA*T86-*gyrA*N90-*par*CI81

TE: Tetracycline, ERY: Erythromycin, CLI: Clindamycin, CIP: Ciprofloxacin, EFX: Enrofloxacin

Figure S1: Alignment between B2 and SE25

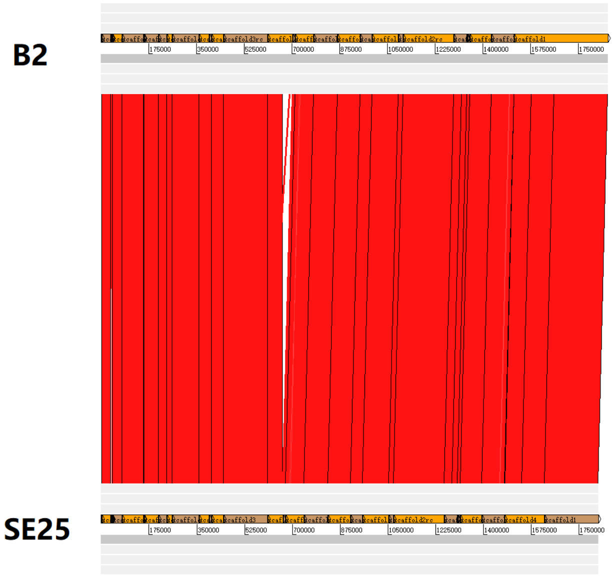

Figure S2: Alignment between B2 and SE27

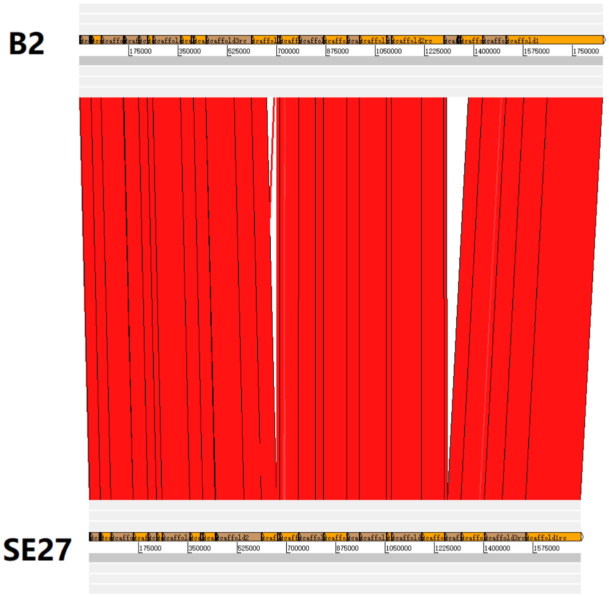

Figure S3: Alignment between B2 and SE-RD

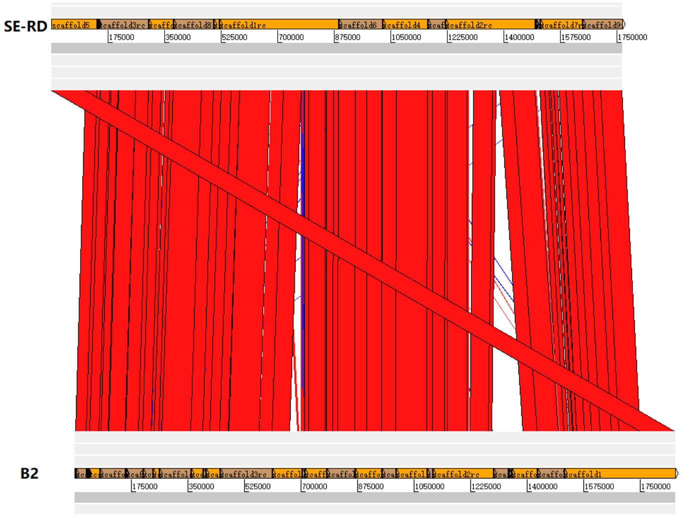

Figure S4: Alignment between B18 and B2

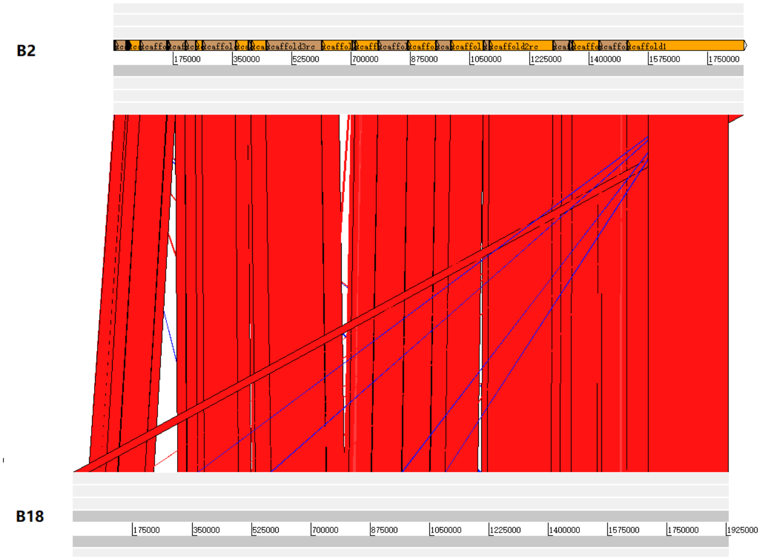

Figure S5: Alignment between B18 and B52

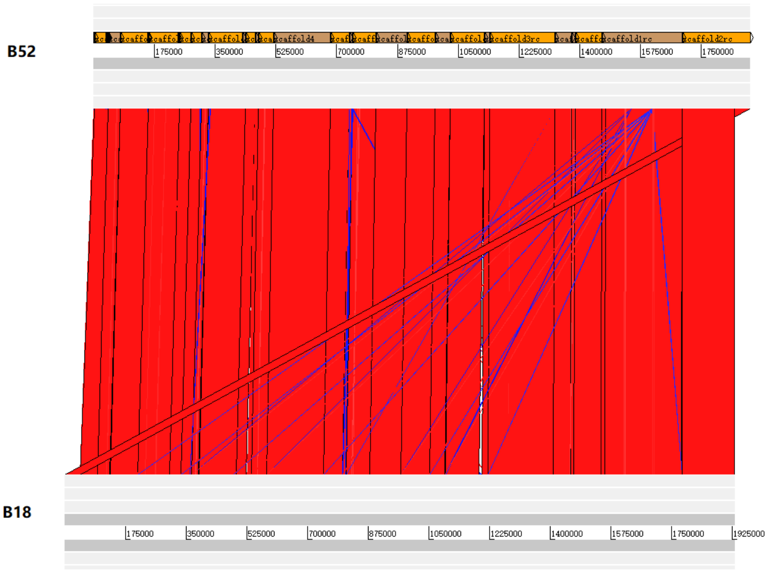

Figure S6: Alignment between B18 and SE25

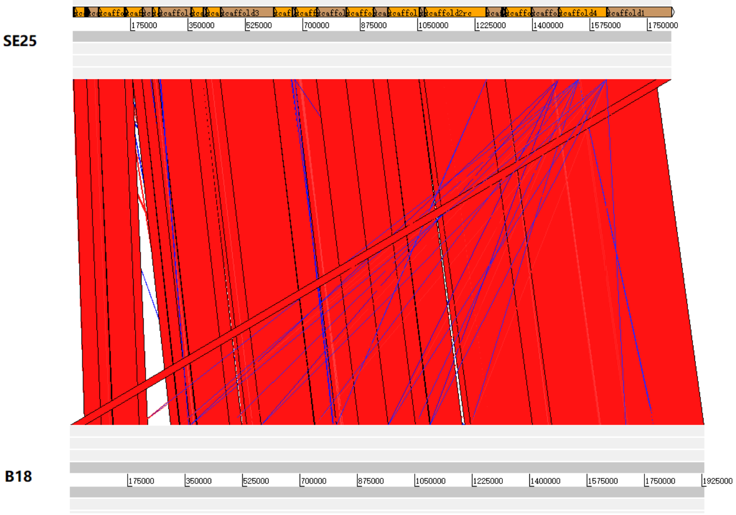

Figure S7: Alignment between B18 and SE27

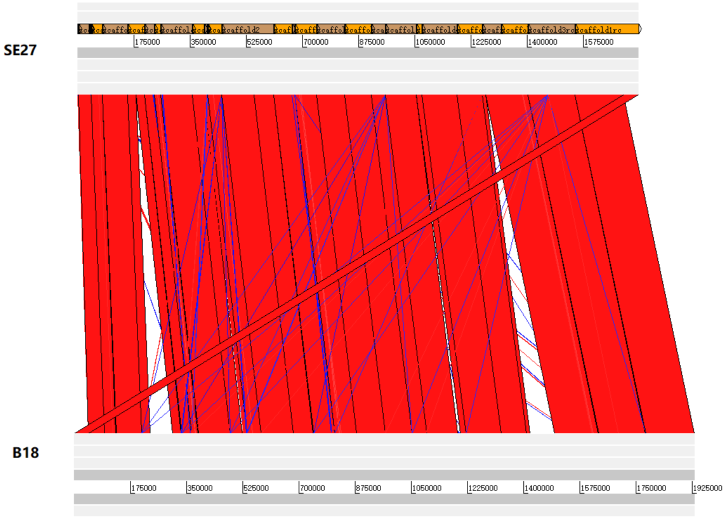

Figure S8: Alignment between B18 and SE-RD

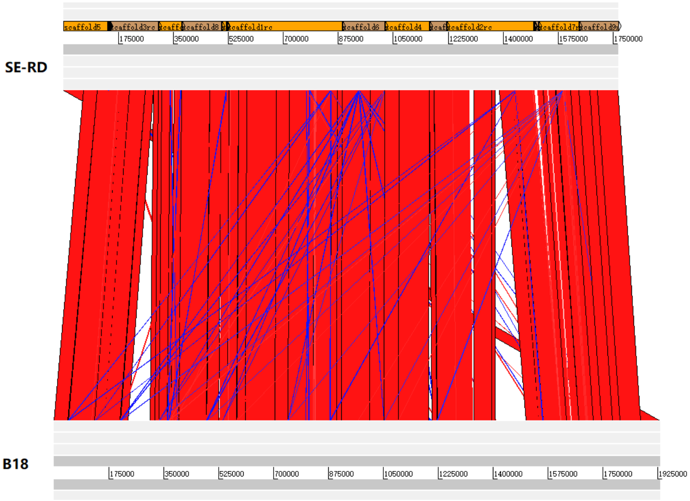

Figure S9: Alignment between B52 and B2

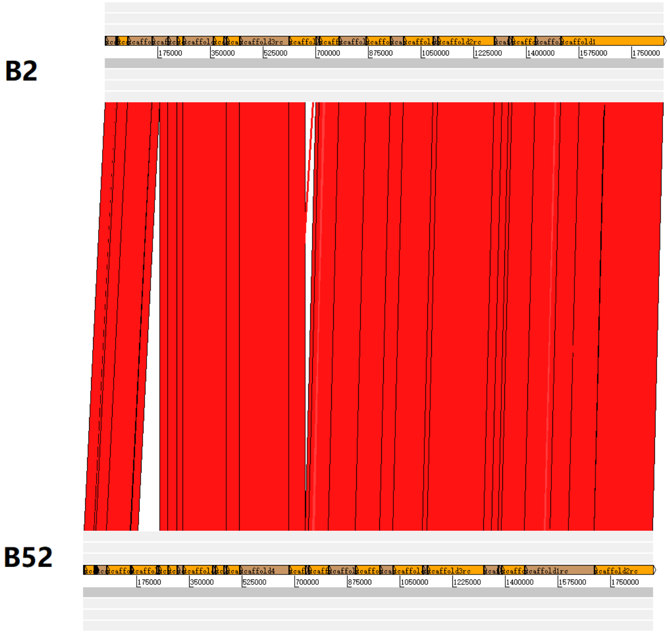

Figure S10: Alignment between B52 and SE25

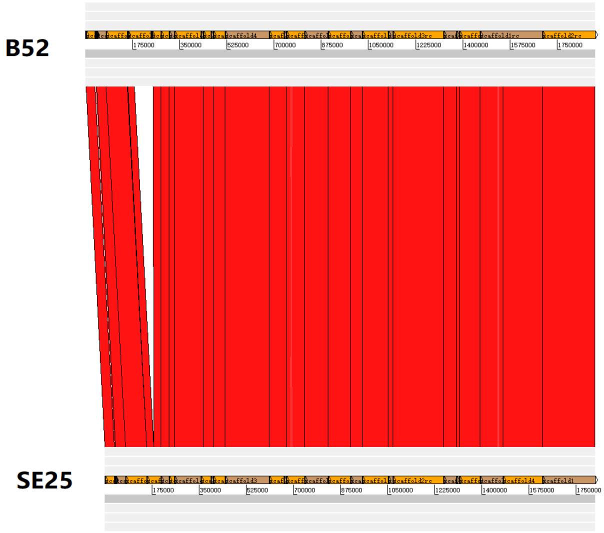

Figure S11: Alignment between B52 and SE27

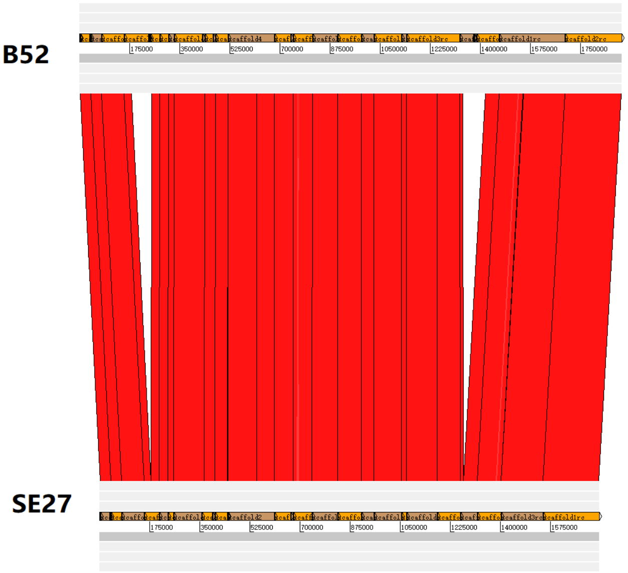

Figure S12: Alignment between B52 and SE-RD

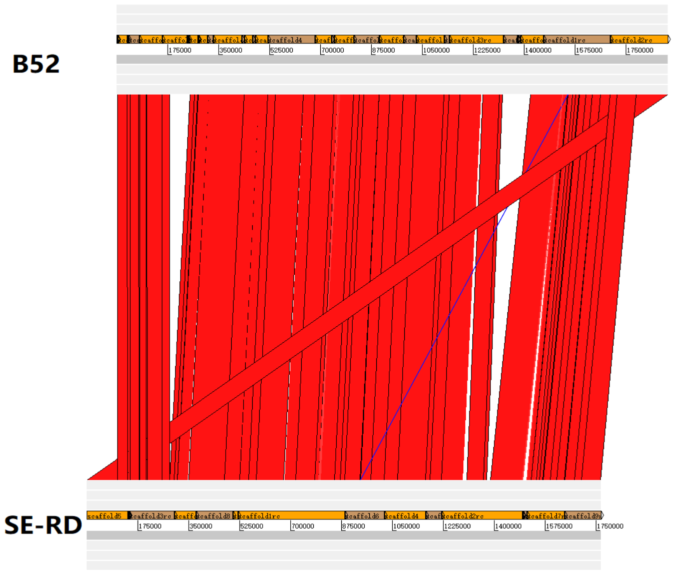

Figure S13: Alignment between SE25 and SE27

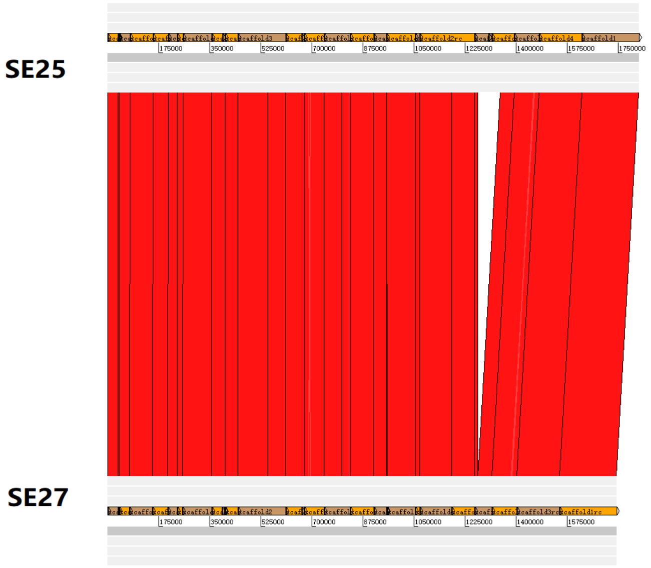

Figure S14: Alignment between SE25 and SE-RD

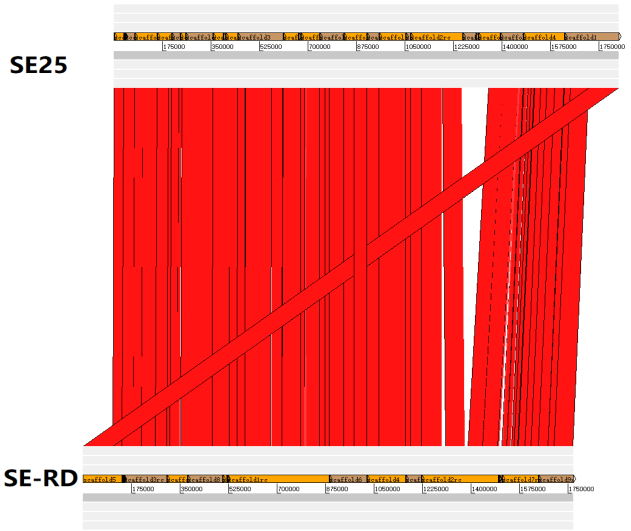

Figure S15: Alignment between SE27 and SE-RD

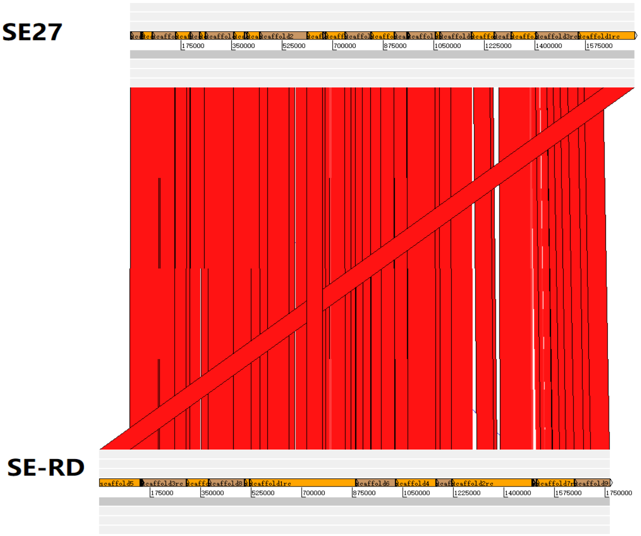

Supplement: Supplementary file 1 [file microorganisms-09-02615-s001.zip › microorganisms-1487578-Supplementary Materials.pdf]
